# Enhanced Availability of Data for Nationally Notifiable Diseases

**Published:** 2014-01-17

Provisional data for cases of selected diseases and conditions reported through the National Notifiable Diseases Surveillance System (NNDSS) by the 50 states, New York City, the District of Columbia, and U.S. territories are collated and published weekly in *MMWR*. Beginning with the January 10, 2014, issue, these data are now available at https://data.cdc.gov in various sortable, machine-readable formats that will make them more usable for analyses. Each weekly issue of *MMWR* includes a link that takes users to that week’s dataset, from which they can link to the new formats.

Previously available options for downloading historical data also continue to be available. For example, a portable document format (PDF) version (and for most issues, a hypertext markup language [HTML] version) of the NNDSS tables for issues from 1994 to the present can still be downloaded from the *MMWR* website (http://www.cdc.gov/mmwr/mmwr_wk/wk_cvol.html), and a tab-delimited text file can be exported from CDC’s WONDER system (http://wonder.cdc.gov/mmwr/mmwrmorb.asp). CDC WONDER contains NNDSS data from 1996 to the present. For older data, complete editions of *MMWR* volumes 1–30 can be downloaded in PDF format from CDC Stacks (http://stacks.cdc.gov). Third-party groups have been able to collect historical NNDSS data from *MMWR* and additional sources. An example of such an external collection is Project Tycho (http://www.tycho.pitt.edu).

Users also should note that a report with final NNDSS data is published in a weekly issue of *MMWR* approximately 8 months after the end of the calendar year. The *Summary of Notifiable Diseases — United States* (http://www.cdc.gov/mmwr/mmwr_nd/index.html) is published annually, approximately 18 months after the end of the calendar year.

